# Cognitive fatigue is related to reduced cerebral perfusion and sustained attention in patients with post COVID-19 condition: An fMRI study

**DOI:** 10.1016/j.ynirp.2026.100406

**Published:** 2026-09-12

**Authors:** Sonia Miri Hedberg, Kristian Borg, Jonas Stenberg, Stina Hedström, Tobias Granberg, Alexandra Gyllenberg, Sven Petersson, Hadrien Van Loo, Love Engström Nordin, Marika C. Möller

**Affiliations:** a Department of Clinical Sciences, Karolinska Institutet, Stockholm, Sweden; b University Department of Rehabilitation Medicine, Danderyd Hospital, Stockholm, Sweden; c Department of Clinical Neuroscience, Karolinska Institutet, Stockholm, Sweden; d Department of Neuroradiology, Karolinska University Hospital, Stockholm, Sweden; e Department of Global Public Health, Karolinska Institutet, Stockholm, Sweden; f Department of Diagnostic Medical Physics, Karolinska University Hospital Huddinge, Stockholm, Sweden; g Department of Neurobiology, Care Sciences and Society, Karolinska Institutet, Stockholm, Sweden

**Keywords:** Post COVID-19 condition, Cognitive fatigability, Psychomotor vigilance test, Fatigue, Functional MRI (fMRI), Arterial spin labeling (ASL)

## Abstract

Persistent fatigue is one of the most prevalent and disabling symptoms of Post-COVID-19 Condition (PCC). To investigate possible mechanisms underlying fatigue in PCC, the present study investigated the relation between different aspects of fatigue and fatigability, and cerebral blood flow (CBF) in individuals with PCC. The participants, 22 patients and 19 controls, matched by age and sex, performed a 20-min-long psychomotor vigilance task (PVT) during fMRI. The total mean reaction time (RT) was used to measure processing speed and fatigability by dividing RTs into quartiles. CBF was measured with pseudo-continuous arterial spin labeling. Self-reported state fatigue (VAS-scale) was assessed before and after the PVT.

Patients reported larger increase in state fatigue (p = 0.004) and longer RTs across all quartiles (p = 0.001) compared to controls, with no difference in fatigability between groups. The voxel-wise analysis of CBF revealed significantly lower global CBF in patients compared with controls (49.2 vs. 51.7 mL/100 g/min). Regional hypoperfusion was observed in several clusters, including the right inferior occipital gyrus (27.9 vs. 46.4 mL/100 g/min), left postcentral gyrus (29.8 vs. 51.3 mL/100 g/min), and right middle cingulate cortex (30.5 vs. 48.7 mL/100 g/min).

## 1 Introduction

Despite the recovery from acute SARS-CoV-2 infection, some individuals develop persistent symptoms, known as Post COVID-19 Condition (PCC). WHO defines PCC with symptoms that last at least two months and persist three months after infection (World Health Organization, 2023). Fatigue and cognitive impairments are among the most common symptoms (Luo et al., 2025) and are related to inability to return to previous work (Jaywant et al., 2024).

Fatigue is multifactorial and is commonly classified as physical, cognitive, or mental (Hu et al., 2024; Wylie et al., 2022). Cognitive fatigue is associated with deteriorating cognitive performance (Wylie et al., 2021) and is often related to slow processing speed in attention demanding tasks (Möller et al., 2014). Also, performance variability has been related to self-rated fatigue in diverse populations (Möller et al., 2017; Walker et al., 2019). Fatigue may present as state fatigue, which is temporary and task-related, or as trait fatigue, a more persistent form often associated with depression, sleep disturbances, or chronic diseases (Behrens et al., 2023). State fatigue is commonly assessed using a Visual analog scale (VAS), whereas trait fatigue is typically measured with questionnaires such as the Multidimensional Fatigue Inventory −20 (MFI) (Smets et al., 1995; Wylie et al., 2022). In PCC, fatigue is often pathological as it does not resolve with rest (Davis et al., 2021) and can impair daily functioning and work capacity (Greenhalgh et al., 2024).

Performance fatigability refers to an objective decline in performance, manifested as slower performance time, increased variability, or increased error rate during physical or cognitive exertion (Behrens et al., 2023; Walker et al., 2019). Performance fatigability is commonly assessed using demanding sustained attention tasks such as the psychomotor vigilance task (PVT) (Behrens et al., 2023; DeLuca, 2005; Lim et al., 2010; Möller et al., 2017). PVT can induce acute mental fatigue through the well-stablished Time-on-Task (TOT) effect, in which subjective fatigue increases and reaction times progressively slow as task engagement continues (Lim et al., 2012). Kluger and coworker's taxonomy conceptualized these terms along two domains. Subjective fatigue is influenced by homeostatic and psychological factors while performance fatigue is linked to biological central and peripheral factors (Kluger et al., 2013).

Research on Corona viruses has raised concerns about the long-term impact of COVID-19 on brain health (Khateb et al., 2020; Möller et al., 2023; Weatherhead et al., 2015). Population-based studies have reported persistent brain changes even after mild infection, including cortical thinning, white matter alterations, and accelerated brain aging associated with cognitive decline (Douaud et al., 2022; Mohammadi-Nejad et al., 2025). Some neuroimaging studies have identified structural changes along with decline in task performance while others have found no significant cognitive deficits (Douaud et al., 2022; Dressing et al., 2022; Fietsam et al., 2023; Guedj et al., 2021). Microvascular alterations have also been proposed as possible mechanisms behind cognitive dysfunction and fatigue in PCC (Möller et al., 2023). Despite these findings, routine clinical MRI sequences typically do not reveal specific intracranial abnormalities in patients with PCC. Cohort studies show that conventional MRI most often appears normal or non-specific findings that are comparable to healthy controls (Vasilev et al., 2023). A study by Kim et al. investigating non-hospitalized individuals recovering from COVID-19 found reduced CBF in the thalamus, orbitofrontal cortex, and basal ganglia. Among participants who experienced long-term fatigue, additional perfusion abnormalities were observed in occipital and parietal cortices (Kim et al., 2023). Similarly, Ajčević et al. (2023) observed CBF reductions in frontal, parietal, and temporal areas (Ajčević et al., 2023). Moreover, Qin et al. reported that individuals with more severe PCC symptoms exhibit a greater CBF reduction in the bilateral superior medial frontal gyrus and insula (Qin et al., 2021). However, none of the studies mentioned above have employed task-based fMRI to clarify underlying mechanisms to behavioral task performance.

As fatigue is a predominant symptom in PCC it is particularly interesting to investigate fatigue and cognition alterations in relation to CBF in the PCC population. PVT has been used in previous ASL studies in healthy participants to induce fatigability. The results have shown CBF alterations in the frontal-parietal attention network, cingulate cortex, thalamus, and basal ganglia during the PVT. PVT decline and increased fatigue perception and have also been associated with post-task hypoperfusion in frontal and parietal regions, indicating metabolic/CBF consequences of sustained cognitive effort (Lim et al., 2012). Similar task-related changes in CBF within attentional and frontal networks, have been demonstrated across/in both mild traumatic brain injury and Parkinson's disease (Liu et al., 2016, 2025).

The pathophysiological mechanisms underlying cognitive fatigue in PCC remain poorly understood. In this study, we sought to identify physiological factors that could guide future interventions by examining self-reported state fatigue, cognitive fatigability, processing speed, and performance variability, and their associations with cerebral blood flow (CBF) in individuals with post-COVID condition (PCC) and non-symptomatic controls.

## 2 Methods

### 2.1 Study design and setting

This is a cross-sectional study conducted at the Cognitive Post COVID-19 Clinic at Danderyd University Hospital, Stockholm, Sweden. The clinic primarily serves patients who are experiencing persistent fatigue and cognitive symptoms, which interfere with functioning and work capacity. Patients were referred by primary care providers, following initial rehabilitation efforts.

### 2.2 Study procedure

The present study was a sub-study of a larger research project investigating the relationship between PCC, cognitive function and fatigue (Clinical Trials NCT06042530). Patients underwent a comprehensive medical evaluation to confirm the diagnosis of PCC according to WHO criteria (World Health Organization, 2021). All participants had completed a neuropsychological assessment at the outpatient clinic 1-3 months prior to the fMRI investigation (results not presented in this report). Patients meeting the inclusion criteria were consecutively invited to this sub-study between April 2022 and January 2024. The control group was recruited as a convenience sample through advertisements.

Our study was approved by the Swedish Ethical Review Authority (reference number 2021-03907, 2022-01215-02) and followed the principles outlined in the Declaration of Helsinki. All participants were provided with both oral and written information about the study and informed consent was obtained.

### 2.3 Participants

Individuals were eligible if they were 25-55 years old, had been referred to the specialized Cognitive Post-COVID Rehabilitation Clinic and had a confirmed COVID-19 infection (verified by Polymerase chain reaction test, rapid antigen test, or antibody testing). The narrow age range was selected to minimize potential confounding effects related to neurodevelopment and aging. Individuals younger than 25 years were excluded because the brain, particularly the frontal lobes, may not yet have reached full structural and functional maturity. Individuals older than 55 years were excluded due to the increased likelihood of age-related neurobiological changes, which could introduce additional variability into the brain measures. All patients had experienced an initially mild acute disease course and fulfilled WHO diagnostic criteria for PCC, with cognitive symptoms and fatigue lasting for at least 3 months that caused activity limitations in daily life.

### 2.4 Exclusion criteria

To avoid factors that could affect the fMRI results, patients with recurrent infection symptoms, persistent cardiopulmonary complications, visual impairments, cognitive deficits related to neurodegenerative or acquired brain injury, sedative medication, present or history of a psychiatric condition such as severe depression, or neuropsychiatric diagnosis such as diagnosed ADHD, autism, or history of burn-out, or substance abuse. Additional exclusion criteria were lack of Swedish language proficiency, left-handedness, and MRI contraindication (metal implants, pregnancy, or claustrophobia). The same exclusion criteria were applied to the control group.

### 2.5 Measurements

**Hospital Anxiety and Depression Scale (HADS):** was used to assess symptoms of anxiety and depression. HADS includes two subscales each consisting of 7 items rated on a 4-points Likert scale scores range from 0 to 21 with higher values indicating greater likelihood of clinical anxiety **(HADS-A)** or depression **(HADS-D)** (Zigmond and Snaith, 1983).

### 2.6 Wechsler Adult Intelligence scale (WAIS)-IV Matrix Reasoning

While education level is often used as a proxy for cognitive reserve, it does not fully capture individual cognitive ability. The WAIS Matrices that measure problem-solving abilities has commonly been used as a measure of cognitive reserve (Lezak, 2012) and was used in the present study as a demographic variable to control for cognitive reserve in patients and controls.

### 2.7 Measurements of fatigue

**Multidimensional Fatigue Inventory (MFI-20):** A 20-item questionnaire that measures fatigue across 5 different subscales, general, physical and mental fatigue, reduced activity and reduced motivation was used to measure trait fatigue. Each subscale consists of 4 questions. The score for every subscale is obtained by summing the score for all 4 items. Higher scores indicate greater fatigue severity (Smets et al., 1995). In the present study, the analyses were limited to general fatigue (MFI-GF) and mental fatigue (MFI-MF). These domains were chosen as they most directly reflect the global experience of general fatigue and its cognitive components (Smets et al., 1995). The MFI-20 questionnaire was compiled during the neuropsychological investigation.

**VAS-f:** State fatigue was assessed using a Visual Analog Scale of Fatigue (VAS-f), a stand-alone measure of self-rated state fatigue. Participants were asked to rate their current level of fatigue by responding to the question: “How fatigued do you feel right now”. The scale ranged from 0 to 100, where 0 corresponded to no fatigue at all and 100 worst possible fatigue (Möller et al., 2017; Nordin et al., 2016). Assessment was conducted immediately before and after the MRI-examination.

**The psychological vigilance task (PVT):** To assess sustained attention and performance fatigability, a reaction time task was implemented using E-Prime software (Psychology Software Tools Inc., Pittsburgh, PA, USA) (Lim et al., 2010; Möller et al., 2017). The task was performed in the MR-scanner. During the task the participants were required to press a button with their right hand as quickly as possible when the target stimulus, a set of four zeros enclosed in a red rectangle, was displayed. Participants were instructed to refrain from responding in case of any other stimulus.

Participants received visual feedback following each response. This feedback was displayed for 1 s. An incorrect response or a response that exceeded 1 s was presented by “false answer” or “no answer” by the system, respectively. The intervals between stimuli varied in pseudo-random manner, ranging from 2 to 10 s. The whole task lasted for 20 min for all participants. The total number of responses ranged from 177 to 213.

For analytical purposes related to performance fatigability, each participant's RTs were divided into four quartiles, based on the response count, with each quartile containing approximately the same number of responses However, due to individual differences in response count and the pseudo-random timing of stimuli, the temporal alignment of these quartiles was less precise than that used in the analysis of regional CBF during the PVT, which was divided into exact 5-min quartiles. Key performance metrics included the average reaction time (RTmean) and the standard deviation of reaction times (RTStd). This last measure served as a normalized index of variability in response speed (Nordin et al., 2016). To quantify fatigue during PVT, two approaches were used. First, fatigue was assessed using a repeated-measures design across the four quartiles. Second, we computed a dichotomized variable by subtracting the mean RT in the last quartile from the mean RT from the first quartile.

### 2.8 MRI procedure

Arterial spin labeling (ASL) is a non-invasive magnetic resonance imaging (MRI) technique that measures CBF (Cerebral blood flow). CBF is closely related to brain metabolism and can serve as a biomarker for brain activity and has shown to be altered in neurological conditions (Ajčević et al., 2023; Möller et al., 2017; Xie et al., 2016).

Before the MRI procedure, participants administered the VAS-before and performed a training 2-min session of the PVT. All scans were obtained on a 3 T MAGNETOM Prisma^Fit^ MRI system (Siemens Healthineers, Erlangen, Germany) equipped with a 64-channel receive-only head coil at Karolinska University Hospital, Stockholm, between 12:00 p.m. and 5:00 p.m. to minimize the circadian effect.

The imaging included a T1-weighted MPRAGE, resting state fMRI sequences before and after PVT, pCASL during PVT, susceptibility-weighted imaging, and T2-weighted FLAIR. The resting-state data has been reported by Hedström et al., (2026); Hedström et al. (2026). The pCASL data was acquired during the PVT using a 2D EPI readout sequence with GRAPPA factor 2, following the pseudo-continuous labeling scheme described by Wu et al. (Wu et al., 2007a). The acquisition parameters were: labeling duration = 1600 ms, post-labeling delay (PLD) = 1000 ms, TE/TR = 12/3500 ms, FOV = 230 × 230 mm^2^, matrix size = 64 × 64, 34 slices of 4 mm thickness, interslice gap of 1 mm, a sampling bandwidth of 3004 Hz/pixel, echo spacing 0.49 ms, and an RF excitation flip angle of 90°. The total sequence duration was 20:27 min (347 measurements). No background suppression was applied. The PLD of 1000 ms is shorter than the 1800 ms currently recommended by ASL consensus guidelines for clinical populations (Alsop et al., 2015). This value reflects protocol choices made when the sequence was originally established for this cohort, and we address the implications of this choice in the Limitations section. No separate M0 calibration scan was acquired; quantification was performed utilizing the control-label time series average according to established toolbox protocols (Wang et al., 2008).

Participants were securely positioned in the head coil using padding to minimize involuntary head movements. Before data acquisition, during the resting-state phase, participants were instructed to direct their gaze toward a white cross displayed on a black screen and to refrain from engaging in specific thoughts. This guidance aimed to maintain participant alertness and reduce visual distractions. The total duration of the effective MRI scanning time was 45:20 min. The participants completed VAS-f again after the procedure. A schematic overview of the study design is provided in supplementary material, Fig. S1.

### 2.9 Image post-processing

All structural imaging was reviewed by a board-certified neuroradiologist. The post-processing of the pCASL data was performed off-line using shell scripts calling C-programs from the AFNI package (Analysis of Functional NeuroImages, http://afni.nimh.nih.gov/afni/). The main preprocessing steps included: (1) motion correction by 3D rigid-body image registration implemented in 3dvolreg; (2) creation of brain mask; and (3) voxel-wise rCBF computation by extraction of even- and odd scans according to previously established equation (Wang et al., 2008). A mean perfusion-weighted image was then generated and used as a reference for spatial registration. For anatomical alignment, the perfusion-weighted image was co-registered to each subject's high-resolution T1-weighted structural image using affine registration (align_epi_anat.py) with a local Pearson correlation cost function optimized for cross-contrast alignment. The T1-weighted images were skull-stripped and intensity-normalized prior to registration.

Subsequently, the T1-weighted images were normalized to standard space using an affine transformation to the Montreal Neurological Institute (MNI) template with 3dAllineate (12-parameter transformation, mutual information cost function). The resulting transformations were concatenated and applied to rCBF maps, thereby resampling the perfusion data into MNI space while preserving alignment with individual anatomy. For the pCASL data translational motion parameters were inspected for all participants to ensure that head displacement did not exceed one voxel size (3.6 mm) to minimize residual motion-related effects not fully corrected by motion correction. For rotation parameters, a corresponding threshold of 0.07 radians was applied, derived by dividing the displacement limit by an assumed brain radius of 50 mm (θ = s/r = 2/50). The general image quality was inspected manually for all acquired sequences to make sure no artifacts affecting image analysis were present. Mean whole-brain gray matter CBF was extracted for each subject by applying a standard MNI gray matter mask to the normalized individual CBF maps.

### 2.10 Statistics

Descriptive statistics and self-reported data were analyzed using IBM SPSS Statistics for Windows, Version 29.0.1.0 (171), IBM Corp, Armonk, NY, USA. Continuous variables, normally distributed, were analyzed using independent-samples Student's t-tests for between-group comparisons and paired student's t-test for within-subject comparisons. For non-normally distributed continuous variables, the Mann-Whitney *U* test was used for between-group comparisons and the Wilcoxon signed-rank tests for within-subject analysis. Categorical variables were compared using Chi-square tests.

A mixed ANOVA was conducted with time (before and after PVT) as a within-subject factor and group (patients vs. controls) as a between-subject factor. Differences between VAS-before and VAS-after the PVT were also computed (VAS-diff). To investigate associations between subjective and objective fatigability, linear regression models were performed with the objective fatigability measure (RTmean) as dependent variable. The independent variables included self-rated fatigability scores (VAS-before, VAS-after, and VAS-diff). Variability was quantified for PVT using the coefficient of variation (CoV), defined as the standard deviation divided by the mean reaction time for each quartile (CoV = SD/mean x 100%). Spatial coefficient of variation of gray matter CBF (spatial CoV = SD(CBF)/mean(CBF) × 100%) was calculated for each subject using the MNI-normalized CBF maps, masked with a common gray matter mask in MNI space as described above. This measure was used as a proxy for arterial transit time heterogeneity in single-PLD ASL data (Mutsaerts et al., 2017).

Regional cerebral blood flow (rCBF) data were analyzed using a mixed-design ANOVA implemented in AFNI (3dANOVA3, model type = 5). The within-subject factor was PVT-time (four quartiles: Q1-Q4), and the between-subject factor was group (PSS vs. Control). Individual subjects were modeled as a random factor to account for repeated measurements. This design allowed us to test the main effects of time and group, as well as their interaction on rCBF.

Voxel-wise linear regression analyses were conducted using AFNI's 3dRegAna to explore the relationship between rCBF and mean reaction time for each quartile of the PVT. Data from all participants across each quartile was pooled for initial analysis, with subsequent regressions performed separately for each group. Regression analyses were also performed with VAS-before, VAS-after and the coefficient of variation (CoV) for the reaction time as variables. Group analysis was also performed using AFNI multivariate modelling (3dMVM) performing an ANOVA with mean reaction time as a variable resulting in RT-effect, group difference and interaction RT x group. 3dMVM is a valuable complement to 3dRegAna because it allows simultaneous modeling of multiple factors and covariates (e.g., group effects and interactions), providing a more flexible and comprehensive framework for assessing CBF–reaction time relationships. This analysis was performed both with absolute CBF maps and with data sets normalized to each subject global CBF. Using normalized CBF data helps control global inter-subject regression analyses. Group differences were finally studied by *t*-test (3dttest++) for validation, where voxel-wise absolute CBF was included for all datasets.

The statistical significance at family-wise error rate (FWER), p < 0.05 for the three-way ANOVA, regression analysis, and *t*-test was assessed by setting a voxel-wise threshold at p < 0.01 and then by imposing a minimum cluster size of at least 30 contiguous voxels. The minimum cluster size was determined from the Monte-Carlo simulation results obtained by using the AFNI program AlphaSim+, which was used to estimate the probability of the random field noise producing clusters of different sizes (Möller et al., 2017). For the simulations, we used the following input parameters: matrix size = 64 × 64 × 34, a gray matter mask based on the MNI template, voxel-wise threshold value p < 0.01, 10^6^ iterations, and Full-Width Half-Maximum (FWHM) = 6.8 mm, which was the estimated average by applying the AFNI program, 3dFWHMx, to the pooled CBF image data. The simulations with AlphaSim + produced a list of FWER p-values corresponding to different cluster sizes, and a larger cluster size corresponds to a lower FWER p-value. For the primary voxel-wise group comparison, a single mean absolute rCBF map averaging the entire 20-min acquisition session was computed for each participant and compared between groups using an independent samples *t*-test (3dttest++). To investigate time-wise changes and interactions during task performance, the pCASL data was additionally split into four 5-min quartiles (Q1–Q4).

## 3 Results

### 3.1 Characterization of the participants

A total of 22 patients and 22 controls were eligible for the study, but three controls were excluded, one due to motion artifact (movement exceeding one voxel size), one after reporting a high level of alcohol consumption the night before, and one after identification of findings consistent with multiple sclerosis during neuroradiological review of the structural MRI. Of the remaining participants, no structural brain abnormalities were detected. Thus, 22 patients with PCC and 19 controls were included in the analysis. Most of the patients were infected during 2020 and 2021, with a mean time of 32.4 months between the infection and the fMRI investigation. The groups did not differ in age or sex, but a significantly higher proportion of controls had university-level education compared to PCC patients (p = 0.01). The two groups were comparable in terms of occupational background. Consistent with this, cognitive assessments revealed no differences between patients and controls in problem-solving abilities (WAIS-IV Matrix Reasoning), used as a proxy for cognitive reserve (Hedström et al., 2026). PCC patients scored significantly higher than controls on both depression and anxiety (p < 0.001) (Table 1).

**Table 1:** Characteristics of participants.

Variable	Patients, (n = 22)	Controls, (n = 19)	P-value
**Age, years, mean (SD)**	44.18 (7.67)	39.16 (10.14)	
**Median (IQR)**	44.00 (37.50–51.25)	38.00 (29.00–50.00)	0.12
**Sex, woman, n (%)**	15 (68.2%)	14 (73.7%)	0.70
**University Education, n (%)**	68.2	100	0.01
**Profession**			
**Health care**	4	7	
**Economy/management**	8	6	
**Engineering/Tech**	2	4	
**Education/social**	3	1	
**Media**	4	0	
**WAIS-IV Matrix reasoning, mean (SD)**	12.9 (2.6)	13.3 (3.2)	0.561
**Time between infection and imaging in months, mean (SD)**	30.95 (7.25)	N/A	
**Year of infection, %**		N/A	
**2020**	54.6		
**2021**	40.9		
**2022**	4.6		
**HADS – depression, median (IQR)**	9.00 (4.50-13.00)^a^	1.00 (0.00-2.00)	< 0.001
**HADS Anxiety, median (IQR)**	7.00 (4.50-11.00)^a^	2.00 (1.00-5.00)	0.001
**MFI-20 General Fatigue, median (IQR)**	18.00 (15.75-20.00)	9.00(6.00-11.00)	< 0.001
**MFI-20 Mental Fatigue, median (IQR)**	16.00 (13.00-18.00)	8.00 (6.00-10.00)	< 0.001

*Note*. HADS, Hospital Anxiety and Depression Scale; IQR, interquartile range; MFI-20, The Multidimensional Fatigue Inventory; PCC, post COVID-19 condition; SD, standard deviation; VAS, Mann-Whitney U tests were used for group comparison. Mann-Whitney *U* test, Students'*t*-test, and Chi-square tests were used for group comparisons.

a one missing.

### 3.2 Self-rated fatigue measures

*Trait fatigue:* Both general and mental fatigue were strongly elevated in PCC patients compared to controls (general fatigue: 18.00 vs. 9.00; mental fatigue: 16.00 vs. 8.00, both p < 0.001) as measured by MFI-20 (Table 1).

*State fatigue:* The time × group interaction effect was significant (p = 0.009). Patients with PCC showed greater increase in state fatigue (VAS-f) than controls (patients: 31.49; controls: 17.70). Patients also had higher VAS-f score at both time points (mean difference before 17.18; mean difference after: 30.97) and between-group difference was larger after the task.

**Fig. 1:**
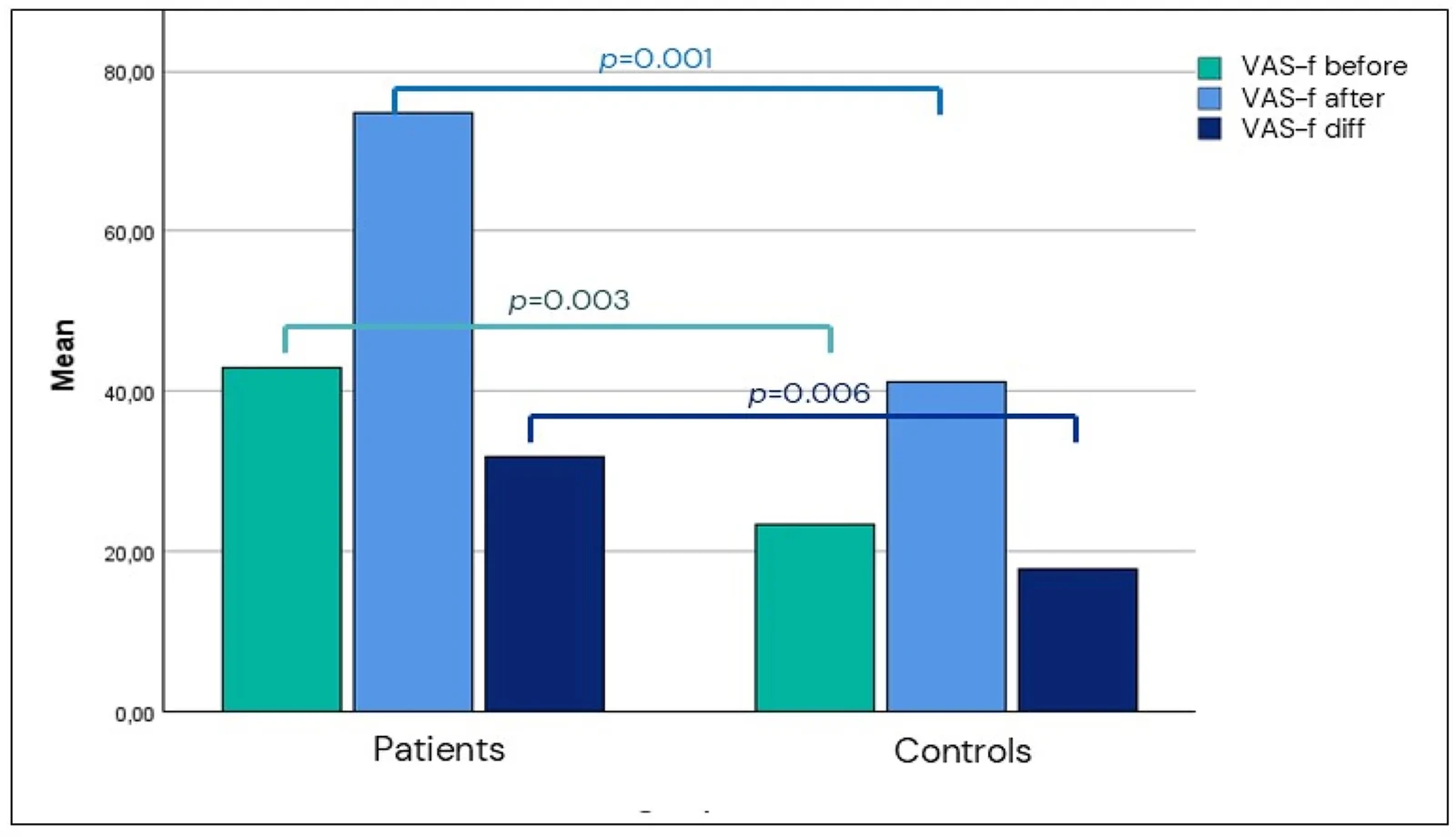
Group differences in VAS-fatigue before and after MRI scanning and difference scores (VAS-diff) **Note.** VAS-fatigue ratings for PCC participants and controls assessed before and after the MRI session, together with VAS-difference scores (VAS-before - VAS-after). PCC showed higher fatigue both and baseline and following scanning, as well as a larger fatigue increase across the session. Abbreviations: VAS, visual analog scale.

Additionally, state fatigue scores among the patients showed greater variability before the task compared to controls (SD = 19.0 vs. 15.7), but the variability in both groups increased after PVT (SD = 21.6 vs. 21.1). Group comparisons using Mann-Whitney *U* test showed statistically significant difference at all time points (p = 0.003 before, p < 0.001 after, and p = 0.006 for the fatigue difference) (Fig. 1, Table 2).

**Table 2:** Group comparison of self-rated fatigue between patients and controls.

Variable	PCC (n = 22)	Control (n = 19)	P-value
**VAS-f before median (IQR)**	4.80 (2.60-5.90)	2.04 (1.1o-3.5)	0.003
**VAS-f after median (IQR)**	8.16 (5.60-9.20)	3.90 (2.00-6.20)	< 0.001
**VAS-f difference median (IQR)**	3.10 (2.00-4.00)	1.30 (1.00-3.00)	0.006

*Note.* IQR, interquartile range; PCC, post COVID-19 condition; SD, standard deviation; VAS, Visual Analogue Scale. Mann-Whitney U tests were used for group comparison.

### 3.3 Performance on the PVT

**Fig. 2:**
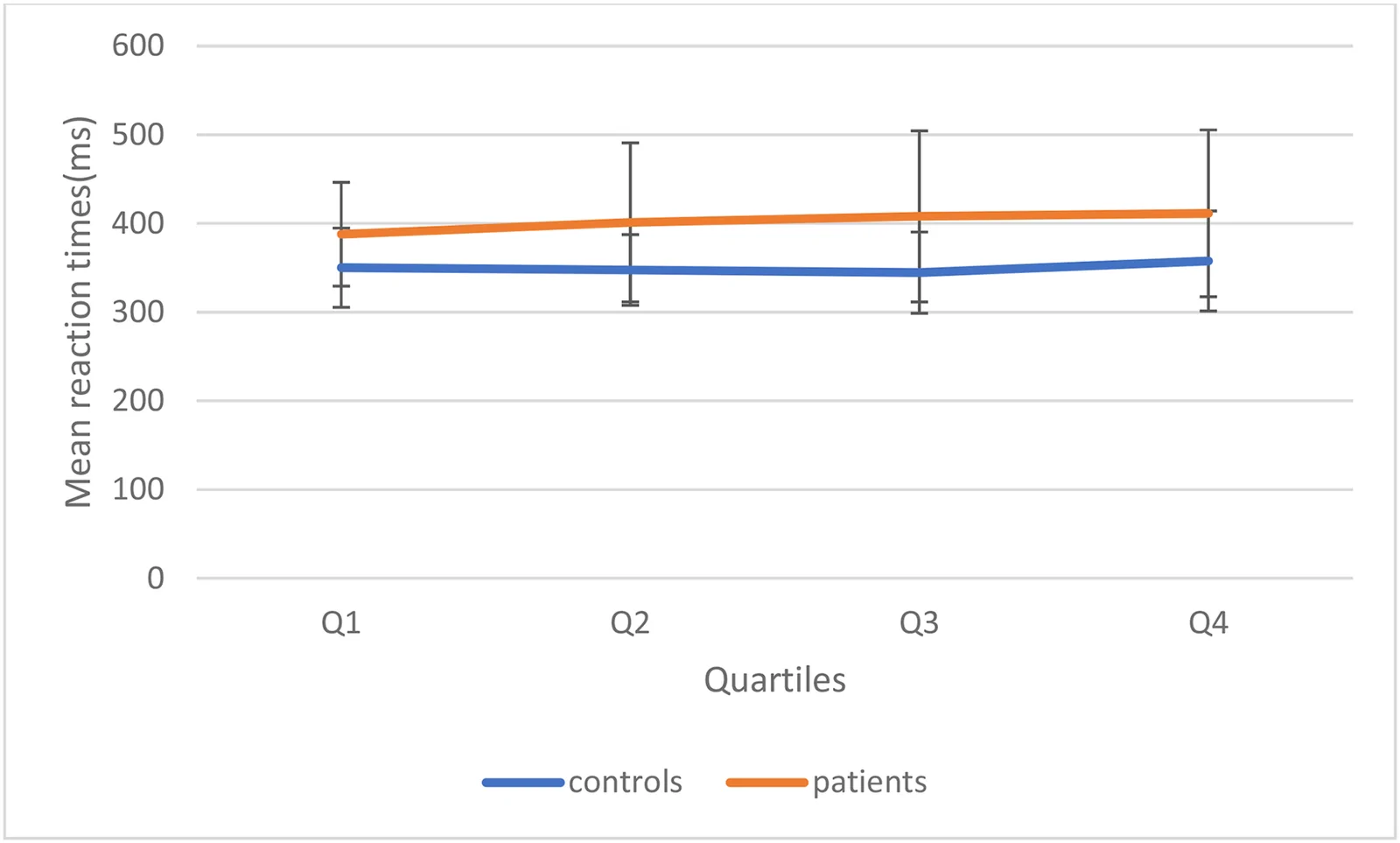
Reaction times across quartiles in patients and controls **Note.** Mean reaction times (ms) across the four PVT (psychomotor vigilance task) quartiles for patients and controls. Patients showed consistently slower responses throughout the task, together with a more pronounced increase in reaction time across quartiles. Error bars represent standard deviations.

RTs were significantly longer in the patient group compared to controls across all quartiles (Table 3 and Fig. 2), with differences increasing between Q2-Q3. Effect sizes were medium to large across all the quartiles and the overall test.

**Table 3:** Variation of performance time as coefficient of SD/mean on the PVT task for patients and controls.

Quartile	Performance-variability – patients (CoV)	Variability difference from Q1 - patients	Performance-variability for controls	Variability difference from Q1 - controls
**Q1**	15,1%	-	12,7%	-
**Q2**	22,3%	47.7	11,5%	−11.5
**Q3**	23,6%	56.3	13,3%	4.7
**Q4**	22,9%	51.7	15,7%	23.6

Abbreviations: Q: quartile.

A mixed ANOVA was conducted with quartiles (Q1- Q4) as a within-subject factor and group (patients vs. controls) as a between-subject factor. The quartile × group interaction was not significant, (F (3, 117) = 0.76, p = 0.520, effect size = 0.019), suggesting that the change overtime did not differ between groups. There was no significant main effect of quartiles (F (3, 117) = 2.40, p = 0.071, effect size = 0.058), indicating that reaction times did not change systematically across quartiles. The main effect of group was significant, (F (1,39) = 5.80, p = 0.021, effect size = 0.129), with PCC patients showing overall slower RTs compared to controls.

Performance fatigability occurred in a comparable proportion of individuals in both groups (59% in PCC and 53% in controls, RT-difference > 0). However, the PCC patients exhibited greater variability in RTs (SD) in every quartile. This variability tended to increase across the first three quartiles (SD_Q1_ = 58.5, SD_Q2_ = 89.7, SD_Q3_ = 96.4, SD_Q4_ = 94.0. The coefficient of variability was relatively equal in both patients and controls in the first quartile but increased with 48% in the second quartile for the patients while the performance variability for the controls remained relatively stable over time (Table 3). There were no statistically significant differences in error rates between the groups.

### 3.4 Associations between fatigue measurements and PVT

For the total group the state fatigue measured *before* PVT was significantly associated with mean RT (Spearman's rho = 0.53 p = 0.02) indicating that higher fatigue before PVT was associated with slower responses. No significant association was observed between state fatigue *after* PVT and mean RT, nor with the subjective state fatigability (VAS-diff) and results on the PVT. To examine whether state fatigue was related to performance variability, Pearson correlations were conducted between CoV and the VAS-f (before, after, and differences). None of the correlations reached significance.

### 3.5 CBF differences

**Fig. 3:**
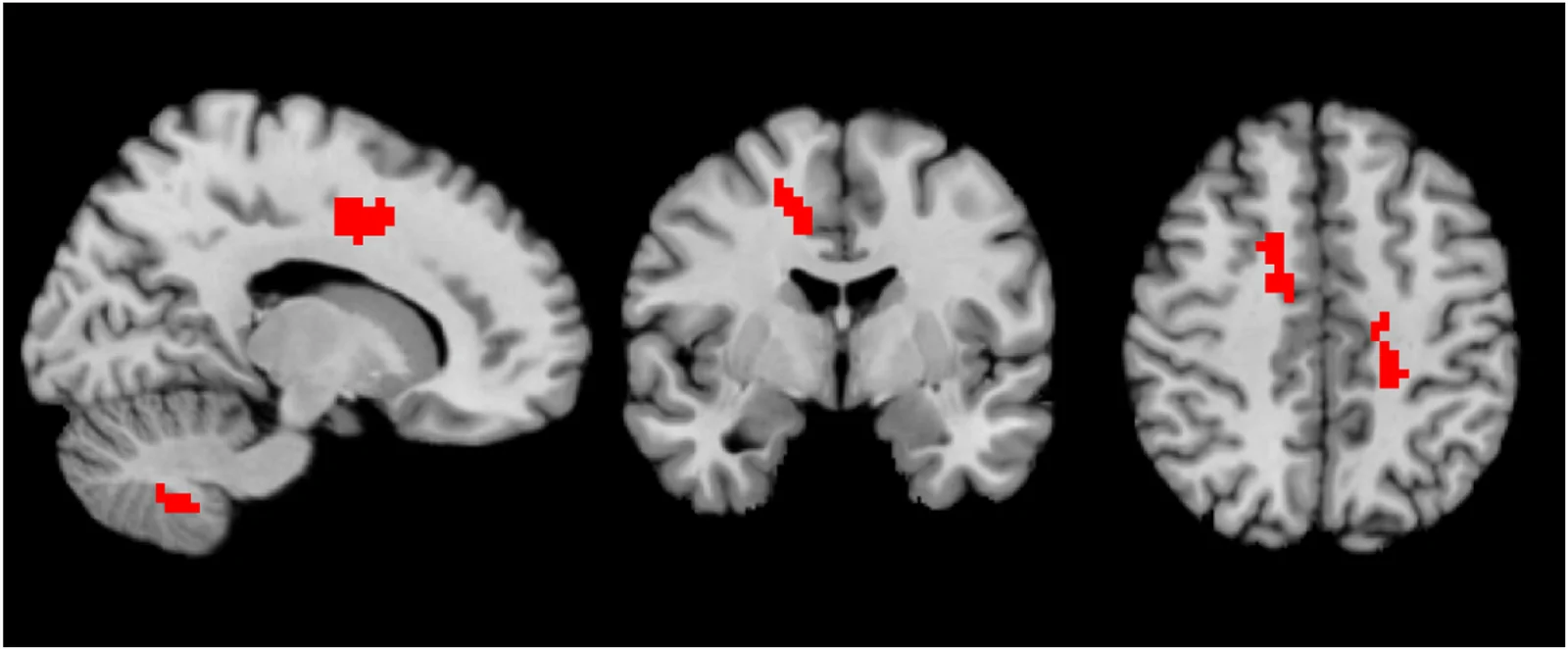
Group differences in cerebral blood flow (CBF) between patients and controls **Note.** Voxel-wise t-test revealed significantly lower CBF in patients compared to healthy controls. Clusters shown in red represent regions where patients exhibited reduced perfusion after corrections for multiple comparisons (p < 0.05, cluster-level). Images are displayed in sagittal, coronal, and axial views on a standard temple.

The *t*-test showed significantly decreased CBF for patients compared to healthy controls in several regions including right inferior occipital gyrus, left postcentral gyrus, right middle cingulate cortex, right cerebellum and left middle occipital gyrus, as detailed in Table 4 and Fig. 3. The group component in the multivariate modelling showed results matching the *t*-test, both with absolute CBF and normalized CBF. In significant clusters, the CBF was on average 38.2% lower in PCC patients compared to controls. While examining whole-brain gray matter, PCC patients showed a 4.8% lower mean CBF (49.3 ml/100 g/min) than controls. The PCC group also demonstrated greater variability (SD 10.7 vs. 8.0), a slightly higher maximum value (77.2 vs. 68.0), and a similar minimum CBF compared to controls (35.5 vs. 35.3 ml/100 g/min). These descriptive statistics show a general whole-brain difference with a wider distribution within the PCC group. To further explore whether group differences in CBF might partly reflect arterial transit time (ATT) effects rather than purely tissue perfusion, we calculated the spatial coefficient of variation (CoV = SD/mean of voxel-wise CBF across gray matter) for each subject as a proxy for ATT heterogeneity. Patients with PCC showed significantly higher spatial CoV than controls (46.4 ± 7.8% vs. 41.2 ± 5.0%; Mann-Whitney U = 305, p = 0.013).

**Table 4:** Results from voxel-wise statistical analysis of calculated CBF-data. A minimum cluster size of 30 contiguous voxels has been applied together with a corrected p-value of 0.01.

Region	Centre of mass (x, y, z MNI coordinates)	CBF Patients (ml/100 g/min)	CBF Controls (ml/100 g/min)	Statistical contrast	Vox/Cluster Volume cm^3^
**Right inferior occipital gyrus**	40, −62, 12	27,9	46,4	*t*-test1	98/2.646
**Left postcentral gyrus**	−23, −32, 39	29,8	51,3	*t*-test2	94/2.538
**Right middle cingulate cortex**	10, −7, 34	30,5	48,7	*t*-test3	62/1.674
**Right cerebellum**	10, −60, −50	38,5	55,2	*t*-test4	35/0.945
**Left middle occipital gyrus**	−23, −55, 31	35,1	60,0	*t*-test5	30/0.810
Right middle cingulate cortex	13, 6, 44	33,1	48,0	ANOVA group	73/1.971
Right caudate nucleus	25, 27, 11	47,1	53,6	ANOVA time	42/1.134
**Global GM CBF**		49,2	51,7		

*Note.* Voxel-wise statistical analysis of calculated cerebral blood flow (CBF) data comparing patients and controls. A cluster threshold of 30 contiguous voxels and a corrected p value of 0.01 were applied. Regions listed show significant group differences or time-by-group interactions. Coordinates are given in MNI space and cluster volumes are reported in cm^3^.

The ANOVA showed significant time effect in the right caudate nucleus. Group effect could be seen in the right middle cingulate cortex. No interaction effect between group and time could be seen (Table 4). The multivariate modelling showed a global effect on RT and interaction RT x group, both with absolute CBF and normalized CBF, also when including global CBF as a covariate, indicating a global correlation between RT and CBF between the groups.

Regression analysis between CBF and individual CoV for patients showed significant correlations to several brain regions (Table 5). The corresponding test for controls did not show any significant results.

**Table 5:** Results from voxel-wise regression analysis of the calculated CBF-data and coefficient of variation (CoV) from the PVT reaction time for patients. A minimum cluster size of 30 contiguous voxels has been applied together with a corrected p-value of 0.01.

Region	Centre of mass (x, y, z MNI coordinates)	#Vox/Cluster Volume cm3
Right/left Cuneus	4, −87, 23	222/8.22
Right Postcentral Gyrus	28, −36, 56	99/3.67
Right Putamen	25, 12, −10	80/2.96
Left Hippocampus	−22, −38, 5	74/2.74
Left Superior Occipital Gyrus	−17, −84, 14	49/1.81
Right Middle Frontal Gyrus	31, 15, 44	46/1.70
Left Superior Temporal Gyrus	−56, −39, 14	41/1.52
Left Cerebellum	−11, −57, −49	40/1.48
Right Angular Gyrus	40, −42, 23	38/1.41
Right Inferior Frontal Gyrus	52, 24, −1	36/1.33

*Note.* Regions listed show significant correlation between CBF and CoV for PVT reaction time for patients. Coordinates are given in MNI space and cluster volumes are reported in cm3.

## 4 Discussion

To identify possible relation between fatigue and underlying physiological factors we examined self-rated state fatigue, state fatigability as well as performance fatigability, processing speed, and performance variability in relation to CBF in patients with PCC and non-symptomatic controls.

In our study, PCC patients reported significantly higher state fatigue, both before and after the PVT and exhibited significantly longer reaction times during the PVT. As to performance fatigability, no significant differences compared to controls were detected in terms of slowing performance time but as performance variability. Perfusion analysis revealed reduced CBF, not only in several regions but also globally across the whole brain and correlated with overall reaction time as well as individual performance variability among the patients indicating a relation between altered cerebral perfusion and overall cognitive performance as well as fatigue.

### 4.1 Self-assessments and vigilance test

According to contemporary theoretical models, task-induced fatigue reflects a dynamic interplay between subjective exhaustion and changes in performance, shaped by psychophysiological factors such as mood, stress, motivation, anxiety and arousal (Kluger et al., 2013). In our sample, both groups showed the expected increase in perceived fatigue following the task. However, PCC consistently reported higher fatigue levels before and after the task, and significantly higher state fatigability despite lack of performance fatigability on the PVT. However, their performance variability increased over time, which is considered an indication of fatigue (Behrens et al., 2023; Walker et al., 2019).

They also showed consistently longer RTs compared with controls. In other conditions such as traumatic brain injuries (TBI), where fatigue is a frequent symptom, it has been related to slower performance time on attention-demanding tests (Johansson et al., 2009; Ziino and Ponsford, 2006). As perceived fatigue and attention processing share underlying brain mechanisms in effort-regulation systems, reduced efficiency and slower information processing system may lead to increased perceived fatigue (Darnai et al., 2023). In this context, slowed processing speed in PCC may reflect underlying homeostatic or physiological mechanisms of fatigue (Davis et al., 2021) contributing to a decline in task performance and reduced quality of life, particularly in individuals with chronic conditions such as PCC (Behrens et al., 2023).

The increased performance variability on the PVT observed in the patient group but not in the control group, represents a novel finding that, to our knowledge, has not been previously reported in studies of PCC – possibly because this measure is seldom examined. Earlier studies on patients with multiple sclerosis and mild TBI – conditions strongly associated with persistent fatigue, have shown that performance variability may be a stronger predictor of self-reported fatigue than fatigability itself (Morrow et al., 2015; Möller et al., 2017). Moreover, there is growing evidence that PCC is not a homogeneous neurobiological condition. Fatigue may arise from diverse underlying mechanisms, including autonomic dysregulation, neuroinflammation, altered neurovascular function, or impaired energy metabolism (Davis et al., 2021). The large standard deviations in fatigue ratings observed in our patient group, compared with controls, support substantial within-group heterogeneity, consistent with earlier neuroimaging studies(Douaud et al., 2022; Guedj et al., 2021).

The prolonged RTs and increased performance variability observed in PCC group may reflect reduced processing efficacy, delayed motor responses, compensatory strategies, or deficits in performance monitoring. The absence of a clear decline in performance over time may be explained by factors such as motivation or stress. In clinical experimental settings, patients may exert a disproportionately high level of cognitive effort to maintain performance during sustained attention tests. Such compensatory effort may temporarily mask performance decline while simultaneously contributing to a greater subjective experience of fatigue. Consequently, post-task recovery may be prolonged or more complex in PCC patients. Therefore, the absence of observable performance decline should not be interpreted as evidence of lack of fatigability. However, firm conclusions cannot be drawn, as a self-reported measure of mental effort was not included in the present study. Future studies should consider assessing cognitive effort and the recovery process in the days following cognitive exertion when interpreting performance outcomes.

### 4.2 Arterial spin labeling

Our voxel-wise analysis revealed hypoperfusion in the right inferior occipital gyrus, the left post-central gyrus, right middle cingulate cortex and the cerebellum, with the most pronounced reduction observed in the middle cingulate cortex – regions that have been associated with fatigue also in healthy individuals (Darnai et al., 2023). These areas are critically involved in visuospatial processing, somatosensory integration, emotional and autonomic regulation, and cognitive control (Ajčević et al., 2023). Together, these findings indicate altered function in neural networks essential for attention and sensorimotor function during PVT. It is worth noting that several significant clusters identified in our analysis extended into or adjacent to white matter (WM) regions, showing apparent CBF values around 50 ml/min/100g (Table 4). Given that true WM perfusion is physiologically much lower, this relatively high value likely reflects a combination of partial volume effects, inherent to the 2D acquisition slice thickness, and macrovascular signal components. Due to the short PLD (1000 ms), a portion of the ASL signal may still reside within larger arterial transit vessels. Consequently, the observed group differences in these areas might not purely reflect tissue hypoperfusion but could instead indicate altered arterial transit times (ATT) or microvascular hemodynamics in PCC patients. Consistent with this, patients showed significantly higher spatial CoV of gray matter CBF than controls, a metric that has been proposed as a proxy for ATT heterogeneity in single-PLD ASL data (Mutsaerts et al., 2017). This finding lends additional support to the interpretation that part of the observed CBF differences may reflect delayed or more heterogeneous arterial transit in PCC patients rather than uniform tissue hypoperfusion alone, although this cannot be definitively confirmed without multi-PLD data. Delayed blood arrival can manifest as an apparent drop in local CBF. Importantly, since the identical acquisition protocol and short PLD were applied systematically to both patients and controls, any potential measurement bias or overestimation of absolute values remains constant across all subjects. The statistically significant group differences observed cannot therefore be dismissed as a methodological artifact, but rather signify a robust, objective divergence in cerebral hemodynamics between PCC patients and controls.

The regions identified with reduced CBF in the present study include areas both within and outside the core fatigue network clusters described by Schumann et al. While the mid-cingulate cortex is considered part of the core fatigue network, the postcentral gyrus and inferior occipital gyrus were not among the clusters identified in their systematic review (Schumann et al., 2025). The cerebellum, however, has been associated with the extended fatigue network, reflecting its important role in motor-cognitive integration (Zhang et al., 2023). It should be noted that this review did not include studies specifically investigating fatigue in PCC. Consequently, the defined clusters and fatigue network are based on evidence from other conditions -such as MS, Chronic fatigue syndrome, Parkinson's disease, and stroke, which limits the extent to which direct comparisons with PCC can be made (Schumann et al., 2025).

Importantly, our multivariate modelling demonstrated that RTs were not only associated with CBF in isolated regions relevant to vigilance performance but were also linked to reduced perfusion at a global level. This association was observed for both absolute and normalized CBF and remained significant even after adjustment for global CBF, indicating that the relationship is not driven by any single brain region. Individuals with PCC exhibited a stronger coupling between lower perfusion and slower as well as more variable RTs compared with the controls, suggesting that attentional systems in PCC may be particularly sensitive to perfusion deficits. Together, these findings imply that hypoperfusion in PCC is not merely a regional phenomenon, but may reflect a broader, system-level vulnerability with functional consequences during attention-demanding tasks.

Kim et al. (2023) investigated COVID-19-related fatigue and reported widespread CBF reductions in subcortical and prefrontal regions, including the thalamus, orbitofrontal cortex, occipital, and parietal cortices, and basal ganglia. No regions showed increased perfusion compared with controls. In fatigue-specific analyses, elevated CBF was observed in superior occipital and parietal regions, alongside decreased CBF in inferior occipital regions. Both their findings and ours implicate occipital regions in COVID-19-related fatigue. However, our results further underscore sustained task-specific hypoperfusion in neural circuits related to attention and motor coordination during task performance (Kim et al., 2023). These perfusion patterns partially overlap with COVID-19-related brain changes, described in FDG-PET studies, which demonstrate diffuse cortical hypometabolism, frontal involvement, and cerebellar hypermetabolism and persistent abnormalities in temporal and insular regions. Taken together, these results show that reduced metabolic and perfusion activity in cortical and subcortical regions may contribute to the experience of fatigue in PCC (Guedj et al., 2021; Karimi-Galougahi et al., 2020).

While our task-based fMRI data did not reveal the compensatory frontal recruitment described by Niemczak et al. (2025), our results converged with theirs in demonstrating associations with perceived fatigue severity. (Niemczak et al., 2025).

The hypoperfusion observed during task performance may reflect a combination of underlying physiological mechanisms, such as systemic inflammation, endothelial dysfunction, neuroimmune activation, and acute resource depletion, induced by cognitive load (Lersy et al., 2022). Our findings support the hypothesis that reduced CBF is associated with underlying microvascular dysfunction and blood-brain barrier (BBB) dysfunction. Zhao et al. (2025) demonstrated that increased microvascular pulsatility, measured by pCASl, was strongly correlated with aging, suggesting that altered perfusion dynamics can serve as a biomarker of microvascular pathology (Zhao et al., 2025). Furthermore, reduced CBF measured by pCASL has been shown to correlate with cognitive impairment in individuals at risk for small vessel disease, supporting its role as an indicator of microvascular dysfunction (Jann et al., 2021). Consistent with this, increased BBB permeability in conjunction with reduced CBF has been observed in stroke-affected regions in a pilot study (Mouchtouris et al., 2024). Additional evidence comes from Tanashyan et al., who used a modified Stroop test combined with BOLD fMRI and reported a greater activation in supramarginal gyri, posterior cingulate cortex, opercular parts of the precentral gyri, and bilateral posterior cerebellar lobes in patients, interpreted as compensatory recruitment(Tanashyan et al., 2024). In contrast, Rubin et al. conducted a cross-sectional MRI study using WEPCAST to assess BBB integrity in individuals with PCC and found evidence of long-term increased permeability, while CBF and brain volume remained unchanged (Rubin et al., 2025).

Within the PCC group, higher local CBF was associated with a higher CoV. This finding may suggest heterogeneity in adaptive neurovascular capacity within the patient population. Patients exhibiting very low variability may not represent a more efficient state, but rather a reduced ability to dynamically adjust cerebral perfusion in response to changing metabolic demands. In contrast, patients with moderately higher local CBF may retain a greater capacity for compensatory cerebrovascular recruitment, resulting in increased performance variability during cognitive engagement. In this context, increased variability could indicate constrained neurovascular responsiveness. Similar interpretations have been proposed in fatigue-related conditions, where variability in CBF has been associated with adaptive capacity rather than dysfunction per se. However, this interpretation remains speculative, and further studies are needed to examine whether variability in CBF reflects available adaptive resources or compensatory capacity in this patient population (Boissoneault et al., 2019; Kutz et al., 2026).

Our findings complement previous evidence showing CBF alteration in PCC. Earlier studies have reported globally reduced CBF in gray matter, with hypoperfusion in frontal, parietal, and temporal regions, and no correlation with MoCA scores. Similarly, we observed a global reduction in CBF, and no regions showing increased perfusion. However, beyond these overall reductions, our results revealed functionally meaningful perfusion abnormalities in occipital and cerebellar regions that were associated with both subjective fatigue and performance on the PVT. This pattern suggests a more localized and functionally relevant hypoperfusion in PCC (Ajčević et al., 2023) which may mirror the cognitive and functional difficulties experienced by patients in everyday lives.

Overall, these findings suggest that pCASL may serve as a biomarker of microvascular changes across a range of conditions. The hypothesis of microvascular involvement in PCC is further supported by converging evidence from related studies. This convergence strengthens the interpretation that CBF alteration observed in PCC may, at least in part, reflect underlying microvascular pathology.

### 4.3 Strengths and limitations

This study has some limitations that should be acknowledged. First, the relatively small sample size (22 PCC patients and 19 controls) limits the statistical power and generalizability of the findings. Secondly, there was a notable difference in educational levels between the two groups, which could have influenced cognitive performance and introduced bias. However, there were no group differences regarding the other proxy measure of cognitive reserve – namely the Matrix reasoning. Thirdly, it was not possible to exclude prior SARS-CoV-2 infection in controls, as public health policy at the time did not support testing for mild symptoms. Instead, efforts were made to ensure that the control participants had not experienced prolonged symptoms following a suspected SARS-CoV-2 infection. Fourthly, a methodological limitation of our study is the use of a relatively short post-labeling delay (PLD = 1000 ms), which falls below the international consensus recommendations for general clinical ASL (Alsop et al., 2015). This protocol choice was not used to optimize SNR according to current recommendations but reflects an outdated PLD setting; we acknowledge this as a limitation. A short PLD increases the risk of arterial transit time (ATT) artifacts, and our finding of elevated spatial CoV in patients is consistent with such effects contributing to the observed group differences. A major strength of our study is the control of potential confounding factors, such as neuropsychiatric conditions and medication use. We ensured a highly homogeneous sample by excluding participants with depression or neuropsychiatric disorders, as well as those using medications known to cause fatigue. This minimizes major physiological influences, although more subtle factors such as motivation or stress cannot be eliminated. All included patients had a verified COVID-19 diagnosis, which further increased homogeneity. Another strength is that all testing was performed at the same time of day for all participants, which is important to limit the effects of circadian rhythms and fatigue fluctuations.

## 5 Conclusions

Individuals with PCC showed pronounced subjective fatigue together with slower and more variable performance on the PVT, consistent with an attenuated capacity to sustain attention. In addition, we observed associations between fatigue measures and reduced cerebral perfusion, both globally and in regions implicated in fatigue-related neural networks. Overall, our findings suggest that impaired cerebral perfusion may contribute to the development of fatigue in PCC patients.

## CRediT authorship contribution statement

**Sonia Miri Hedberg:** Writing – review & editing, Writing – original draft, Visualization, Validation, Software, Project administration, Methodology, Investigation, Formal analysis, Data curation, Conceptualization. **Kristian Borg:** Writing – review & editing, Methodology, Funding acquisition, Conceptualization. **Jonas Stenberg:** Writing – review & editing, Methodology, Investigation, Data curation, Conceptualization. **Stina Hedström:** Writing – review & editing, Investigation. **Tobias Granberg:** Writing – review & editing, Investigation. **Alexandra Gyllenberg:** Writing – review & editing, Investigation. **Sven Petersson:** Writing – review & editing, Investigation. **Hadrien Van Loo:** Investigation. **Love Engström Nordin:** Writing – review & editing, Visualization, Validation, Supervision, Software, Methodology, Investigation, Formal analysis, Conceptualization. **Marika C. Möller:** Writing – review & editing, Writing – original draft, Resources, Project administration, Methodology, Investigation, Funding acquisition, Conceptualization.

## Funding

This research was supported by a grant from Promobilia. No additional external funding was received.

## Declaration of competing interest

The authors declare the following financial interests/personal relationships which may be considered as potential competing interests:Marika Moller reports financial support was provided by Promobilia Foundation. If there are other authors, they declare that they have no known competing financial interests or personal relationships that could have appeared to influence the work reported in this paper.

## Data Availability

Data will be made available on request.
