# Supplementary material for: Cognitive fatigue is related to reduced cerebral perfusion and sustained attention in patients with post COVID-19 condition: An fMRI study
Source: Neuroimage Rep. 2026 Sep 12;6(4):100406. doi: 10.1016/j.ynirp.2026.100406 (PMC13586861; doi:10.1016/j.ynirp.2026.100406)
Supplement: Supplementary file 1 [file mmc1.docx]

**Supplementary material**

**Figure S1.** Overview of study protocol

***Note*** *Schematic illustration of the study procedures. Participants rated state fatigue (VAS-f) before entering the MRI scanner, followed by a resting-state BOLD acquisition. They then completed the 20-minute psychomotor vigilance test (VT) with pseudo-continuous arterial labeling (pCASL). A second resting-state BOLD scan was performed, and the session ended with a post-MRI state fatigue (VAS-f) rating*
***Abbreviations****: BOLD, Blood Oxygenation Level Dependent; fMRI, functional Magnetic Resonance Tomography; PVT, Psychomotor vigilance task; VAS-f: visual analog scale-fatigue;*
